# Quantifying the Financial Value of Clinical Specialty Choice and Its Association With Competitiveness of Admissions

**DOI:** 10.7759/cureus.13272

**Published:** 2021-02-10

**Authors:** Pranav Puri, Natalie Landman, Robert K Smoldt, Denis Cortese

**Affiliations:** 1 Alix School of Medicine, Mayo Clinic, Scottsdale, USA; 2 Center for Healthcare Delivery and Policy, Arizona State University, Phoenix, USA

**Keywords:** undergraduate medical education, graduate medical education, specialty choice, usmle step 1, primary care, net present value

## Abstract

Background

The factors influencing medical student clinical specialty choice have important implications for the future composition of the US physician workforce. The objective of this study was to determine the career net present values (NPVs) of US medical students’ clinical specialty choices and identify any relationships between a specialty’s NPV and competitiveness of admissions as measured by the US Medical Licensing Examination (USMLE) Step 1 scores.

Methodology

NPVs were calculated using the results of the 2019 Doximity Physician Compensation report, a survey of 90,000 physicians. Mean USMLE Step 1 scores for matched US allopathic seniors in the 2018 National Resident Matching Program were used as a measure of clinical specialties’ competitiveness of admissions. We calculated a composite measure of NPV and annual work-hours by dividing each specialty’s NPV by the reported average number of hours worked per year.

Results

In our analysis, orthopedic surgery had the highest NPV ($10,308,868), whereas family medicine had the lowest NPV ($5,274,546). Dermatology and plastic surgery had the highest mean USMLE Step 1 scores (249 for both), whereas family medicine had the lowest (220). Clinical specialties’ NPVs were positively associated with mean USMLE Step 1 scores (Pearson’s r = 0.82; p < 0.001).

Conclusions

In this study, we describe associations suggesting that medical students respond to financial incentives in choosing clinical specialties and that these decisions are mediated by USMLE Step 1 scores. This underscores the importance of titrating and aligning incentives to improve the allocation of medical students into clinical specialties.

## Introduction

Globally, public opinion polls rank medicine as the most respected and trusted profession [1]. Physicians are uniquely positioned within society to alleviate suffering and improve the health of others. Therefore, medicine has long been thought of as a calling, rather than a profession. Psychologists Dik and Duffy describe a calling as a career that has an external summons, provides a sense of meaning or purpose, and is used to help others in some capacity [2]. In addition, physicians who identify with medicine as a calling are less likely to experience burnout [3].

On the other hand, a career in medicine is also financially rewarding. Although medical school tuitions and physician debt burdens have increased markedly in recent years, physicians remain among the highest paid professionals [4]. From a financial perspective, a student’s decision to pursue a medical education can be considered an investment in human capital. Just as a firm’s physical capital comprises buildings and machines, a physician’s human capital comprises medical knowledge and clinical skills. If medical education is an investment in human capital, then medical students may maximize financial returns on their medical education in the same manner that firms maximize returns on physical capital.

Prior research has evaluated careers in medicine as investments in human capital. Marcu et al. [5] and Doroghazi and Alpert [6] have shown that a medical degree confers considerable economic benefit, despite high upfront costs. However, physician earnings vary broadly among clinical specialties. In 2019, the average salary of a neurosurgeon was $617,000, whereas the average salary of a family medicine physician was $242,000 [7]. Similarly, the duration of training also varies widely among clinical specialties. Neurosurgeons complete seven years of postgraduate medical education, whereas family medicine physicians complete three years [8]. Therefore, the financial investment in a career in neurosurgery is markedly different from that of a career in family medicine.

However, if medical students are primarily motivated by a calling, then they likely will choose clinical specialties on the basis of their academic interests or passions. If medical students select clinical specialties purely on the basis of their interests and those interests are diverse, the investment value of a clinical specialty should not be associated with the competitiveness of admission into the field. For example, if neurology is no less “interesting” than orthopedic surgery, admissions for orthopedic surgery programs should be no more competitive than for neurology programs, even if a career in orthopedic surgery is a more financially rewarding investment.

In the United States, graduating medical students apply to residency programs through the National Resident Matching Program (NRMP). During the past 10 years, the NRMP has become increasingly competitive [9], with the average number of residency program applications per US medical graduate increasing from 32 to 60 [10]. In addition, because of the increased adoption of pass/fail grading, US Medical Licensing Examination (USMLE) Step 1 scores are the only non-demographic continuous variable by which residency program directors can screen applicants [11]. Faced with a growing number of applications, residency program directors have increasingly filtered applicants by Step 1 score, and in a 2018 national survey of program directors, Step 1 score was the most commonly cited factor in choosing candidates to interview [11]. On February 12th, 2020, the National Board of Medical Examiners (NBME) and the Federation of State Medical Boards (FSMB) announced that USMLE Step 1 will transition from a three-digit numeric score to a pass/fail outcome, potentially as soon as 2022 [12]. However, until this change takes effect, program directors will likely continue to use USMLE Step 1 scores to filter applicants.

Therefore, the existing literature and trends described above raise the question: do medical students choose clinical specialties as a calling or as an investment in human capital [13-14]? Further, how is this decision influenced by USMLE Step 1 performance? Although previous studies have attempted to answer these questions using surveys, stated preferences often differ considerably from the actual choices made by individuals.

In this study, we calculated the investment (net present) values of careers in various clinical specialties. We then evaluated the associations among these net present values (NPVs), annual work-hours, and competitiveness of admissions as measured by USMLE Step 1 scores. Our aim was to generate hypotheses and facilitate further discussion on these dimensions of medical education.

An earlier version of this article was posted to the Medrxiv preprint server on March 18, 2020.

## Materials and methods

We restricted our analysis to specialties that offered at least 100 positions in the 2018 Accreditation Council for Graduate Medical Education match [9] and estimated the investment value of careers in various clinical specialties by using an NPV method [5]. NPVs are frequently used by businesses to project and compare the profitability of different investment options [15]. The concept of NPV is best explained through an example. Suppose a firm needs to make a payment of $100,000 exactly two years from now. If a bank offered the firm a 5% interest rate on deposits, then the firm could deposit $90,702.95 ($100,000/1.05^2^) today, and in exactly two years, they would have a balance of $100,000. Thus, the NPV of the payment is $90,702.95.

We calculated NPVs by assuming that students enter medical school at age 24 (the mean age of matriculating allopathic medical students [16]) and graduate from medical school at age 28. Residency training lengths were determined from the Washington University St. Louis residency roadmap application [8]. We assumed a postgraduate year (PGY) 1 salary of $55,000 with a 3% straight-line increase in annual salary for the duration of the training period. Our assumed PGY 1 salary is comparable to the median PGY 1 salary reported in the AAMC 2018 Debt Fact Card [16]. We assumed a 3% annual salary growth rate, as this corresponds to a rate of 1% above inflation. We assumed that upon completion of residency, physicians earned their specialty’s national average salary, as reported by the 2019 Doximity Physician Compensation survey of 90,000 physicians [7]. From this point through retirement at age 65, we assumed a 3% straight-line salary increase for the duration of the physician’s career. We assumed a discount rate of 5% throughout the analysis.

We explore the relationship between NPV and admissions competitiveness across clinical specialties. We used clinical specialties’ mean USMLE Step 1 scores for matched US allopathic seniors as a proxy for admissions competitiveness. We descriptively plot mean USMLE Step 1 scores against NPV and estimate correlation using a Pearson’s correlation.

We then analyze how this relationship is affected by accounting for a clinical specialties’ annual work-hours. We cite clinical specialty annual work-hours reported by Leigh et al. [17], who surveyed 6,381 physicians. Work-hours included all medical activities including direct patient care, administrative tasks, and professional duties. We calculated a composite measure of NPV and work-hours. The composite measure was calculated by dividing each specialty’s NPV by the reported average number of hours worked per year. We illustrate the association between mean USMLE Step 1 scores and annual work-hours−weighted NPV and calculate a Pearson’s correlation.

All analyses were carried out using JMP, version 14 (SAS, Cary, NC, USA) and evaluated at a significance level of 0.05. This study was exempt from IRB review as we utilized publicly available datasets that do not contain patient health information.

## Results

The results of our analysis are summarized in Table 1. Orthopedic surgery had the highest NPV ($10,308,868), whereas family medicine had the lowest NPV ($5,274,546). Differences in NPV were driven by differences in average annual salary and by duration of training-physicians with longer training periods receive postgraduate salaries for longer periods than their peers in shorter training programs. Dermatology had the highest annual work-hours−weighted NPV (4,388 [$/annual work-hours]), whereas family medicine had the lowest weighted NPV (2,190 [$/annual work-hours]). Dermatology and plastic surgery had the highest mean USMLE Step 1 scores (both 249), whereas family medicine had the lowest mean score (220).

**Table 1:** Summary statistics. NPV, net present value; USMLE, US Medical Licensing Examination ^a ^Pathology was excluded because it was not reported by Leigh et al. [17], nor was it included in the 2019 Doximity Physician Compensation Report [7] ^b ^Leigh et al. [17] did not report annual work-hours for radiology and anesthesiology because of high variability

Specialty^a^	2019 USMLE Step 1 score, mean	NPV	Annual work-hours	Annual work-hours−weighted NPV, $/annual work-hours	2019 Average salary	2019 Matched graduates, No.
Dermatology	249	$9,088,220.69	2,154	4,219.23	$450,000	497
Plastic surgery	249	$7,724,423.85	2,471	3,126.03	$433,000	172
Orthopedic Surgery	248	$10,308,868.00	2,715	3,797.00	$526,000	752
Otorhinolaryngology	248	$7,578,424.84	2,524	3,002.55	$398,000	328
Radiation Oncology	247	$9,198,312.29	2,463	3,734.60	$486,000	179
Neurosurgery	245	$10,192,309.02	2,770	3,679.53	$617,000	231
Radiology^b^	240	$8,149,067.01	…	…	$429,000	1,099
General surgery	236	$7,670,463.90	2,826	2,714.25	$403,000	1,432
Internal medicine	233	$5,740,034.35	2,609	2,200.09	$264,000	8,278
Emergency medicine	233	$7,263,452.26	2,205	3,294.08	$336,000	2,458
Anesthesiology^b^	232	$8,199,759.92	…	…	$405,000	1,827
Neurology	231	$6,185,915.53	2,415	2,561.46	$303,000	874
Obstetrics and gynecology	230	$6,817,709.85	2,778	2,454.18	$335,000	1,392
Pediatrics	227	$4,872,532.48	2,212	2,202.77	$223,000	2,867
Psychiatry	226	$5,751,556.94	2,209	2,603.69	$281,000	1,720
Physical medicine and rehabilitation	225	$6,422,838.40	2,157	2,977.67	$315,000	459
Family medicine	220	$5,274,545.54	2,500	2,109.82	$242,000	3,827

**Figure 1:**
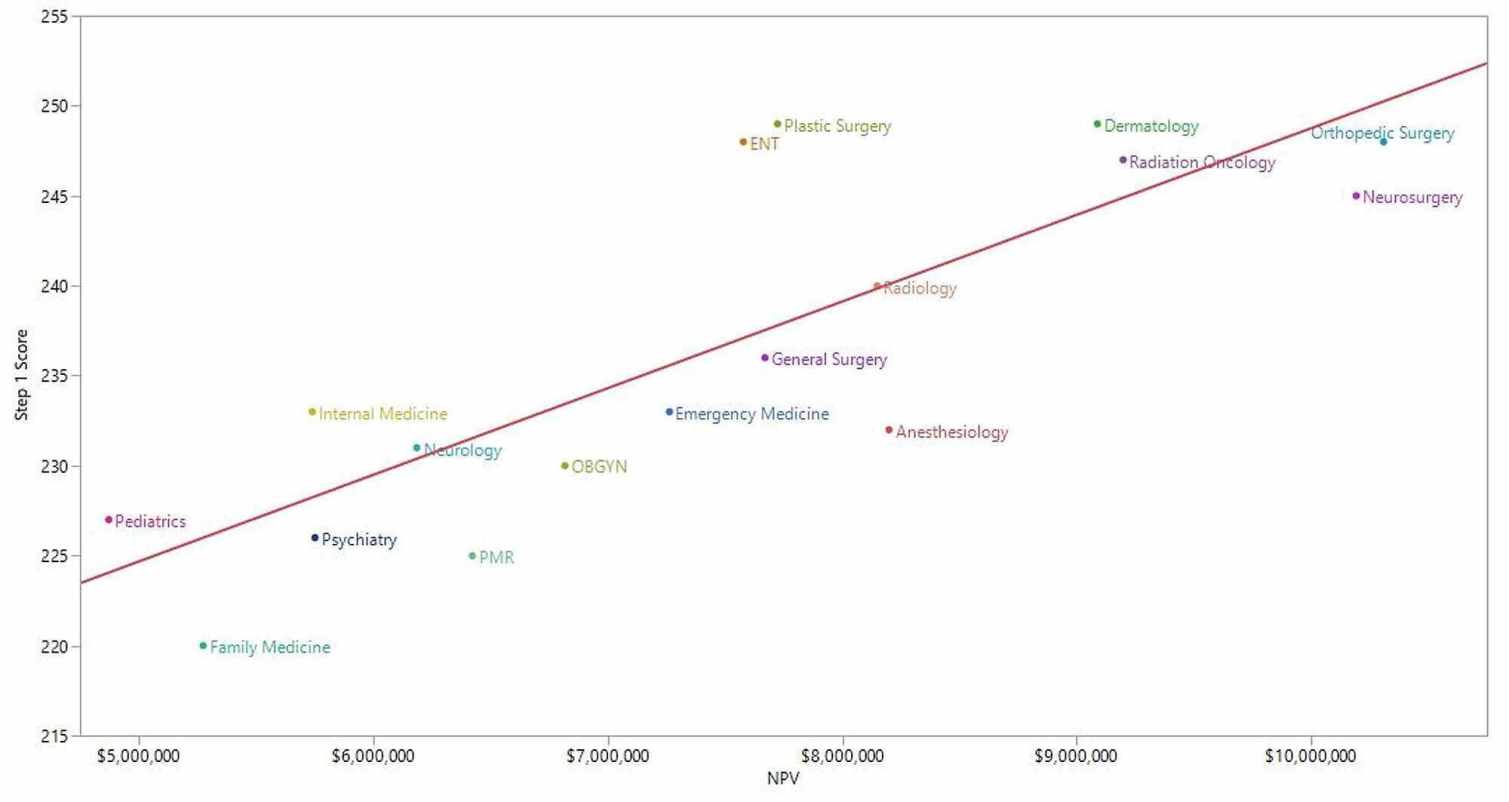
Clinical specialty NPV and mean USMLE Step 1 score. ENT, otorhinolaryngology; NPV, net present value; OBGYN, obstetrics and gynecology; PMR, physical medicine and rehabilitation; USMLE, US Medical Licensing Examination Pearson’s r = 0.82; p < 0.001

**Figure 2:**
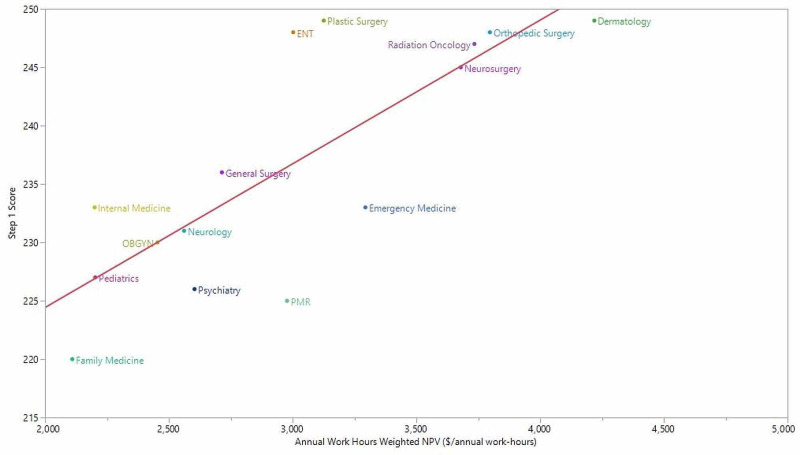
Clinical specialty annual work-hours−weighted NPV and mean USMLE Step 1 score. ENT, otorhinolaryngology; NPV, net present value; OBGYN, obstetrics and gynecology; PMR, physical medicine and rehabilitation; USMLE, US Medical Licensing Examination Leigh et al. [17] did not report annual work-hours for radiology and anesthesiology because of high variability

We observed a positive association between a clinical specialty’s mean USMLE Step 1 score and its NPV (Pearson’s r = 0.82; p < 0.001) (Figure 1). Similarly, USMLE Step 1 scores were positively associated with the composite measure of NPV and annual work-hours (Pearson’s r = 0.79; p < 0.001) (Figure 2).

The association between a clinical specialty’s mean USMLE Step 1 score and the specialty’s number of matched graduates was not statistically significant (Pearson’s r = -0.47; p = 0.06). However, after the exclusion of internal medicine as a potential outlier, we observed a statistically significant negative association (Pearson’s r = -0.75; p < 0.001). With the exception of internal medicine, the clinical specialties with the fewest available training positions had the highest mean USMLE Step 1 scores. Similarly, we observed a negative association between clinical specialty NPV and the specialty’s number of matched graduates (Pearson’s r = -0.55; p = 0.02).

## Discussion

In this study, we calculated the NPV of careers in various clinical specialties and evaluated the associations between NPV, the number of annual work-hours, and USMLE Step 1 scores. Our calculations show wide disparities in the NPVs of clinical specialties, with careers in primary care (family medicine, general internal medicine, pediatrics) having the lowest NPVs. In addition, we identified a positive correlation between a clinical specialty’s NPV and its mean USMLE Step 1 score. In other words, the clinical specialties with the highest NPVs were also the most difficult to gain admission into. Similarly, we observed a positive association between a clinical specialty’s annual work-hours−weighted NPV and mean USMLE Step 1 score. These associations, at least in part, suggest that medical students respond to the financial incentives of the residency admissions process.

Medical students in the United States spend a substantial portion of time preparing for USMLE Step 1, but this is a rational response to the incentives they face [18]. To the degree USMLE Step 1 scores serve as gatekeepers to higher NPV specialties, the financial consequences of a student’s USMLE Step 1 score could amount to millions of dollars. Students who obtain high USMLE Step 1 scores are able to clear the cut-off scores applied by program directors and remain competitive for a broad range of clinical specialties. However, students with lower USMLE Step 1 scores may not meet score cut-offs and are screened out from more competitive, high NPV clinical specialties. This restricts students with lower USMLE Step 1 scores to less competitive, lower NPV clinical specialties. Therefore, students with lower USMLE Step 1 scores may not be able to gain admission into the clinical specialties they are genuinely interested in. To the same end, students with higher USMLE Step 1 scores may forego clinical specialties of genuine interest in favor of pursuing higher NPV clinical specialties.

To this end, the decision of NBME and FSMB to move to pass/fail score reporting for USMLE Step 1 has the potential to improve the allocation of physicians into clinical specialties. However, when USMLE Step 1 score reporting transitions to pass/fail, residency program directors will continue to receive increasing numbers of applications, but will be confronted by a paucity of objective, standardized measures to screen applicants. Some have suggested that this will cause USMLE Step 2 Clinical Knowledge scores to assume the current role of USMLE Step 1 scores [19]. Yet, regardless of changes to the admissions process, there will remain wide variations between the NPVs of clinical specialties. These variations influence medical student specialty choice and create strong financial incentives for medical students to pursue fields outside of primary care.

This study has several limitations. Because this study used an observational design, we are not able to draw inferences about the causality of the relationships we observe. Confounding factors could influence the relationships we observe. For example, clinical specialties with fewer residency positions had higher USMLE Step 1 scores and higher NPVs. However, the purpose of this study was simply to describe associations between NPV and the competitiveness of residency admissions, which have not been reported in the literature to date. Similarly, we calculated NPVs by assuming physicians earned their clinical specialty’s mean salary, with 3% straight-line growth. However, salaries within clinical specialties can differ broadly, depending on numerous factors such as sub-specialization, geographic location, and practice in an academic or non-academic setting. In addition, this study did not account for factors that may influence specialty choice, including prestige, outstanding debt, age, race, and gender. Future studies could leverage an experimental design or novel econometric techniques to elucidate the mechanisms underlying the associations described in this paper.

## Conclusions

In this study, we describe associations suggesting that medical students respond to the financial incentives of clinical specialty choice and that these decisions are mediated by USMLE Step 1 scores. These associations have important implications for policy-makers, physicians, and medical students alike. The US healthcare system faces a significant and growing shortage of primary care physicians. At the same time, our calculations show careers in primary care have the lowest NPVs. Therefore, this study underscores the importance of titrating and aligning economic incentives to improve the allocation of medical students into clinical specialties.
